# McGill University

**Published:** 1896-05

**Authors:** 


### McGILL UNIVERSITY.

THE annual meeting for the conferring of degrees in comparative medicine and veterinary science was held in the William Molson Hall, McGill University, on March 27th, and was presided over by Mr. J. R. Molson. Among the governors present were, including Principal Peterson and Sir William Dawson, Professor Clark Murray, Mr. S. Finley, Dr. W. I. Shaw, Mr. Justice Davidson, Mr. Justice Archibald, Dr. Craik, dean of the medical faculty, Professors Wesley Mills, Girdwood, McEachran, dean of the faculty of comparative medicine and veterinary science; Dr. Charles McEachran, Professors Baker, Penhallow, and Adami, Mr. J. Fleet, Drs. Deeks, Morrow, Johnson, Ruttan, Martin, Mr. W. C. McDonald, Rev. J. Clarke Murray, and Mr. C. J. Gould. Drs. A. W. Clement, of Baltimore, and J. M. Parker, of Haverhill, Mass., two graduates of the University, also occupied seats on the platform.

After prayer by Rev. J. Clarke Murray, Principal McEachran read the reports of the sessions of 1895 and 1896, after which he read the list of those who had passed the examinations in the order of merit.

*Graduating Class.* James E. Craik, Harri H. Dell, John Greer, Charles H. Higgins, Fred. W. Kee, Samuel Macnider, John J. McCarry, Edward H. Morris, John A. Ness, Samuel C. Richards, and Ernest C. Thurston.

*Prize-winners.* Veterinary Medicine and Science, H. H. Dell; Anatomy, R. G. Matthews; Cattle Pathology, H. H. Dell; Cynology, H. H. Dell; Pharmacology and Therapeutics, H. H. Dell; Botany, W. B. Wallis; Chemistry, B. B. Killam; Physiology, B. A. Sugden. For the best general examination in all subjects, silver medal, H. H. Dell.

Extra prizes were also awarded as follows:

For the best essay read before the Veterinary Medical Association: 1st, H. H. Dell. Second and third prizes are added together and divided between Messrs. Kee, Higgins, and Ness.

For the best essay read before the Society for the Study of Comparative Psychology: R. G. Matthew; 2d, H. H. Dell; 3d, F. W. Kee. The first-year prize was won by J. P. Spanton.

Professor Adami's prize of $50 for original pathological research, open to students in the final years in human and comparative medicine, divided between C. H. Higgins, B.Sc., Comp. Med., and Mr. R. H. Martin, Human Med., Mr. Higgins's work being on an epizoötic of chicken cholera near Montreal.

Acting Secretary Brakenridge then administered the oath to the graduating class, and was followed by Principal Peterson, who performed the capping.

The valedictory address was delivered by Harri H. Dell, who, at considerable length and in eloquent terms, traced the history of veterinary science from the earliest stages down to the discovery of printing, when it had received a great stimulus. He also expressed the hope that a post-graduate course would be established by McGill University at an early date. In conclusion, Dr. Dell referred to the graduating class of 1895–96, and proffered some good advice to the students now entering upon the study of their profession.

Professor Wesley Mills[1] replied to the valedictory to the graduates.

Principal Peterson then made a few remarks referring to the excellent addresses of Dr. Dell and Professor Mills, and in the name of the professors of the University tendered his good wishes to the graduates for their future success in life; also expressing the hope that the desire of Principal McEachran for closer affiliation with McGill would be realized, and said he had no doubt the University would do justice in extending the work of the college.

The proceedings closed with benediction by Dr. Shaw.

